# MicroRNA profiles for different tissues from calves challenged with *Mycoplasma bovis* or challenged with *Mycoplasma bovis* and bovine viral diarrhea virus

**DOI:** 10.1371/journal.pone.0271581

**Published:** 2022-07-21

**Authors:** Eduardo Casas, Shollie M. Falkenberg, Rohana P. Dassanayake, Karen B. Register, John D. Neill

**Affiliations:** Ruminant Diseases and Immunology Research Unit, National Animal Disease Center, Agricultural Research Service, United States Department of Agriculture, Ames, Iowa, United States of America; University of Illinois, UNITED STATES

## Abstract

The objective was to determine differences in microRNAs (miRNAs) counts in several tissues of calves challenged with *Mycoplasma bovis* (*M*. *bovis*) or with *M*. *bovis* and bovine viral diarrhea virus (BVDV). Eight calves approximately 2 months of age were randomly assigned to three groups: Control (CT; n = 2), *M*. *bovis* (MB; n = 3), and Coinfection (CO; n = 3). On day 0, calves in CO were intranasally challenged with BVDV and calves in MB with *M*. *bovis*. On day 6, CO calves were challenged with *M*. *bovis*. Calves were euthanized 17 days post-challenge and serum (SER), white blood cells (WBC), liver (LIV), mesenteric (MLN) and tracheal-bronchial (TBLN) lymph nodes, spleen (SPL), and thymus (THY), were collected at necropsy. MiRNAs were extracted from each tissue from each calf. Significant (P< 0.01) differences in miRNAs expression were observed in SER, LIV, MLN, TBLN, SPL, and THY. There were no significant (P> 0.05) miRNAs in WBC. In SER, the CO group had levels of miR-1343-3p significantly higher than the CT and MB groups (P = 0.0071). In LIV and SPL, the CO group had the lowest counts for all significant miRNAs compared to CT and MB. In TBLN, the CT group had the highest counts of miRNAs, compared to MB and CO, in 14 of the 21 significant miRNAs. In THY, the CO group had the highest counts, in 4 of the 6 significant miRNAs compared to CT and MB. BVDV was associated with reduction of miRNAs in LIV, SPL, MLN, and TBLN, and *M*. *bovis* reduced counts of miRNAs in only TBLN. Measuring circulating miRNAs to assess disease condition or to develop intervention strategies to minimize respiratory diseases in cattle caused by BVDV or *M*. *bovis* will be of limited use unless an alternative approach is developed to use them as indicators of disease.

## Introduction

Bovine respiratory disease complex (BRDC) involves different viruses and bacteria including Bovine Viral Diarrhea Virus (BVDV) and *Mycoplasma bovis*. It has been estimated BRDC costs the cattle industry approximately $1 billion annually and is considered one of the most economically devastating diseases that affects the cattle industry [1, 2]. Given that producers in the United States have limited ability to use antibiotics as a preventive measurement to control BRDC, the response of the animal to the pathogen should be evaluated, instead on continuing to focus on the pathogen [3].

Bovine viral diarrhea is a disease caused by one of three *Pestiviruses* species, comprised by bovine viral diarrhea virus 1 (BVDV-1), bovine viral diarrhea virus 2 (BVDV-2), and Ho-Bi-like virus. Financial losses are experienced by producers due to the conditions produced by these viruses [4, 5]. Bovine pestivirus infections can be associated with respiratory, digestive, or reproductive signs, and the infections are usually subclinical [6, 7]. A common characteristic shared among bovine pestiviruses is replication in tissues from organs responsible for the immune response. Replication in immune system can lead to a reduction in circulating lymphocytes and immunomodulation that may be associated with immune suppression and increased susceptibility to secondary infections [8–11].

*Mycoplasma bovis* is an important pathogen that causes respiratory disease in cattle [12, 13], which is the most common pathogen retrieved from lungs of cattle affected with bovine respiratory disease complex. However, additional species of Mycoplasmas are also retrieved [14]. Cattle affected with *M*. *bovis* are usually chronically affected, unresponsive to treatment, and unable to attain commercial weights.

Studies have proposed microRNAs (miRNAs) as biomarkers and as indicators of exposure to pathogens [15–17]. MiRNAs are small non-coding RNAs that alter transcription by inhibiting translation or degrading messenger RNA [18, 19]. There have been several attempts to identify miRNAs associated with BVDV infection [20–22], and with *M*. *bovis* [23]. However, there has been no attempt to establish miRNA profiles in several tissues of animals co-infected with bovine viral diarrhea virus (BVDV) and *M*. *bovis*; therefore, the objective of this study was to determine differences in miRNA counts in several tissues of calves challenged with *M*. *bovis*, or with *M*. *bovis* and BVDV.

## Materials and methods

### Experimental design

#### Animal welfare

Animals used in the study were handled in accordance with the Animal Welfare Act Amendments (7 U.S. Code §2131 to §2156) and all procedures were approved by the Institutional Animal Care and Use Committee of the National Animal Disease Center. Animals were euthanized with intravenous Sodium Pentobarbital per label dose or discretion of the attending veterinarian.

#### Challenge study

Initially, eleven Holstein male calves approximately 2 months of age were randomly assigned to one of four groups: Control (CT; n = 2), bovine viral diarrhea virus (BV; n = 3), *M*. *bovis* (MB; n = 3), and Coinfection with BV and MB (CO; n = 3). On day 0, calves in groups CO and BV were intra-nasally challenged with BVDV and group MB with *M*. *bovis*. Calves in the CT group were given 5 mL of cell culture supernatant of uninfected cells. On day 6, CO calves were challenged with *M*. *bovis*. At the end of the study, it was determined that all calves, except for those in the BV group, were naturally infected with *M*. *bovis* prior to the start of the experiment, determined by antibody measurement using ELISA. To avoid a confounding effect in miRNA expression, it was determined to exclude the BV group from further analysis.

The BVDV isolate used for challenge was a typical virulent BVDV-2, subgenotype A (RS886). RS886 was isolated at the National Animal Disease Center from a clinically normal persistently infected calf [8].The BVDV was propagated in bovine turbinate cells (Btu) that had been tested and found free of BVDV and HoBi-like viruses as previously described [24]. Viral titers were determined via dilution on Btu cells [25]. Endpoints were determined based on immunoperoxidase staining using the monoclonal antibody N2, which binds the E2 protein of BVDV-2 used in the study [26, 27].

*Mycoplasma bovis* isolate KRB5, cultured in 2014 from the lung of a calf with pneumonia [28], was used in this study. KRB5 was grown for 18 to 24 hours at 37°C in an atmosphere of 5% CO_**2**_ in PPLO broth (BD Diagnostic Systems) supplemented with 10 g/L of yeast extract (BD Diagnostic Systems) and 20% horse serum (Life Technologies). Inoculum was prepared as previously described [29], and adjusted to a final concentration of 2 x 10^**10**^ cfu/mL. Each calf intranasally received 5 mL of inoculum containing a total of 1 x 10^**11**^ cfu.

Calves were euthanized 17 days post-challenge and serum (SER), white blood cells (WBC), liver (LIV), mesenteric lymph node (MLN), tracheal-bronchial lymph node (TBLN), spleen (SPL), and thymus (THY), were collected at necropsy. Serum samples were collected from all calves via jugular venipuncture in SST vacutainer tubes (BD, Franklin Lakes, NJ). White blood cells were collected by venipuncture in PAXgene tubes (PreAnalyliX GmbH, Hombrechtichon, Zurich, Switzerland). All samples were stored in RNAlater (Millipore Sigma, Darmstadt, Germany) and stored at -80°C until processed.

### MicroRNA isolation

Total RNA was purified from all tissues using the MagMAXTM mirVanaTM Total RNA Isolation Kit (Life Technologies, Carlsbad, CA, United States) and was eluted in 100 uL of RNase-free water. The concentration and quality of small RNAs in each sample was determined using a 10–40 nucleotide gate on an Agilent 2100 Bioanalyzer Small RNA chip (Agilent Technologies, Santa Clara, CA, United States).

### Library preparation and sequencing

For each tissue, six microliters (6 uL) of small RNA from each extraction was used to prepare individual libraries using the NEBNext Multiplex Small RNA Library Prep Kit (New England BioLabs, Ipswich, MA, United States) and 11 Illumina indexed primers, giving each sample a unique identifier (barcode). Library concentration and purification was performed using the QIAquick PCR purification kit (QIAGEN, Germantown, MD, United States). Each library was run on an Agilent 2100 Bioanalyzer High Sensitivity DNA chip (Agilent Technologies, Santa Clara, CA, United States) to determine quality and quantity of the prepared library between 135 and 170 base pairs. Then, 30 ng of each library was pooled (11 libraries in the pool) and size selected using AMPure XP beads (Beckman Coulter, Indianapolis, IN, United States). Following size selection, library pools were concentrated using the QIAquick PCR purification kit (QIAGEN, Germantown, MD, United States) and eluted in RNase-free water. An Agilent 2100 Bioanalyzer High Sensitivity DNA chip (Agilent Technologies, Santa Clara, CA, United States) was used to determine the concentration of each library pool between 135 and 170 base pairs. The library pool was stored at -20°C until sequencing. The size selected library pool was sequenced as single-end 50 base pair reads using the Illumina HiSeq 3000 System (Illumina, San Diego, CA, United States).

### Data and statistical analysis

FastQC v0.11.21 and fastx_clipper program in a fastx toolkit2 were used to determine the quality of the sequences and remove the adapter sequence from each read, respectively. Unique sequences were collapsed using a custom script and sequences 18–40 nucleotides in length were retained for analysis [30]. These sequences were initially mapped to the *Bos taurus* genome (ENSEMBL UMD3.1.75) using NovoAlign software (Novocraft Technologies), allowing two mismatches. *B*. *taurus* genome aligned sequences were then aligned to a database containing different annotated genome features in order to determine the aligned sequences’ origin: miRNA sequences were downloaded from the website; mitochondrial tRNA, cDNA, and other non-coding RNA sequences were downloaded from ENSEMBL version 75. The sequences that aligned to miRNAs or their flanking sequences were further characterized. These sequences were aligned to a *B*. *taurus* miRNA database (www.mirbase.org) using BLASTN and the results were processed using a custom script. After miRNA sequences were determined, the number of sequences per sample per tissue was obtained using a custom script, and normalization of library size to reads per million (RPM) was obtained for statistical analysis [31]. Sequences are available on the NCBI SAR under BioProject accession number PRJNA530924.

Correlations were obtained using the procedure CORR of SAS (SAS Inst., Cary, NC). The total count number of each miRNAs within each tissue was used. Correlations were determined by comparing among tissues. Data were analyzed with the MIXED procedure of SAS (SAS Inst., Cary, NC). For each miRNA, the model included the effect of treatment (CT, MB, and CO). An overall P< 0.01 was considered statistically significant. Nominally significant (P< 0.05) results were included only when significant (P< 0.01) miRNAs were identified in other tissues. The present study was designed to ascertain nominal significant differences with the minimal number of experimental units. For this reason, it was deemed relevant to present un-corrected P-values. Significances should be taken into consideration when interpreting results.

## Results

### Number of sequences

A total of 1,324,236,847 sequences were mapped to bovine miRNAs (S1 Table). The percentage of miRNAs for SER, WBC, LIV, MLN, TBLN, SPL and THY, were 0.3%, 18%, 7%, 20%, 17%, 18%, and 20% respectively. This corresponds to 368 miRNAs in which at least one of the seven tissues had 1,000 counts or more. The number of miRNA sequences among animals ranged from 90,974,181 to 155,801,395. The average number of miRNA sequences for animals in the CT group was 127,374,259 sequences, while the average for MB and CO was 112,720,346, and 139,397,020 sequences, respectively.

### Correlations among tissues

Table 1 shows the correlations of miRNA expression among all tissues used in the study. Expression of miRNAs in SER is moderately correlated with expression of miRNAs in WBC and LIV; however, the correlation of SER with MLN, TBLN, SPL and THY, is low and statistically near zero. The expression of miRNAs in WBC was uncorrelated with the expression of miRNAs in any of the tissues from the lymphatic system organs. Expression of miRNAs in LIV was moderately correlated with expression of miRNAs in MLN, TBLN, SPL, and THY. There were high correlations (r> 0.85) of expression of miRNAs among tissues from the lymphatic system organs collected except for the correlation between SPL and THY (r = 0.65). Tissues from the lymphatic system organs used in the study seem to have similar expression of miRNAs, which is different from the expression of circulating miRNAs.

**Table 1:** Correlations of number of copies including all miRNAs used in the study, among tissues.

Tissue^a^	SER	WBC	LIV	MLN	TBLN	SPL	THY
SER	1	0.36	0.49	0.18	0.17	0.08	0.17
WBC		1	0.05	0.05	0.05	0.05	0.08
LIV			1	0.77	0.75	0.60	0.68
MLN				1	0.99	0.86	0.87
TBLN					1	0.91	0.85
SPL						1	0.65
THY							1

^a^ SER = Serum; WBC = White blood cells; LIV = Liver; MLN = Mesenteric lymph node; TBLN = Tracheal-bronchial lymph node; SPL = Spleen; and THY = Thymus.

### Significant miRNAs

Table 2 shows the significant miRNAs (P< 0.01) among treatments. Forty miRNAs had differences in expression in all tissues but WBC. Tissues with the fewest significant miRNAs were SER, LIV, and MLN, compared to SPL, TBLN, and THY, which had the greatest number.

**Table 2:** Tissue, miRNA, total number of copies, significance (p-value), normalized mean in reads per million (RPM) by treatment, and standard error (SE).

					Treatment^b^		
Tissue^a^	miRNA	N	P-value	CT	MB	CO	SE
SER	miR-1343-3p	4,233	0.0071	828^d^	770^d^	1,215^c^	58
LIV	miR-382	3,566	0.0040	49^c^	40^c^	28^d^	2
	miR-154b	1,419	0.0064	21^c^	19^c^	9^d^	2
	miR-493	14,120	0.0045	184^c^	143^d^	121^d^	10
SPL	miR-30d	2,639,840	0.0064	13,571^c^	13,625^c^	8,474^d^	800
	miR-126-3p	1,662,798	0.0072	8,321^c^	8,638^c^	5,405^d^	500
	miR-181b	170,422	0.0095	821^c^	912^c^	540^d^	57
	miR-30f	122,556	0.0031	575^c^	637^c^	379^d^	30
	miR-370	23,597	0.0095	162^c^	115^c^	59^d^	13
	miR-421	5,794	0.0038	28^c^	33^c^	20^d^	2
	miR-378b	2,699	0.0098	15^c^	13^c^	9^d^	1
MLN	miR-147	7,161	0.0037	44^c^	17^e^	29^d^	3
	miR-378	996,716	0.0075	4,800^c^	3,945^d^	3,116^e^	300
TBLN	miR-199a-3p	2,851,229	0.0087	18,977^c^	9,155^d^	11,660^d^	1,600
	miR-125b	612,615	0.0059	5,674^c^	518^d^	1,519^d^	600
	miR-24-3p	508,903	0.0055	3,786^c^	1,654^d^	1,666^d^	270
	miR-127	453,851	0.0093	2,740^c^	1,755^d^	1,507^d^	170
	miR-125a	211,721	0.0044	1,536^c^	707^d^	685^d^	100
	miR-23a	189,549	0.0023	1,256^c^	714^d^	670^d^	60
	miR-195	126,986	0.0014	897^c^	426^d^	435^d^	45
	miR-411a	92,960	0.0095	639^c^	278^d^	336^d^	50
	miR-381	66,350	0.0001	451^c^	246^d^	206^d^	12
	miR-224	25,617	0.0036	165^c^	101^d^	84^d^	9
	miR-409a	12,101	0.0064	88^c^	41^d^	35^d^	7
	miR-193b	6,120	0.0083	39^c^	26^d^	18^d^	3
	miR-376d	1,194	0.0076	9^c^	4^d^	4^d^	1
	miR-1468	27,550	0.0026	199^c^	119^d^	77^e^	12
	miR-671	3,532	0.0013	12^d^	13^d^	20^c^	1
	miR-32	120,966	0.0087	350^d^	625^c^	684^c^	45
	miR-29d-5p	9,324	0.0097	25^d^	50^c^	50^c^	4
	miR-21-5p	37,078,062	0.0023	126,876^e^	162,380^d^	208,554^c^	8,000
	miR-2284z	11,073	0.0036	33^e^	50^d^	60^c^	3
	miR-6529a	6,984	0.0051	22^d^	44^c^	31^d^	2
	miR-96	3,250	0.0039	7^e^	26^c^	15^d^	2
THY	miR-200b	77,991	0.0047	225^d^	149^d^	571^c^	57
	miR-147	3,256	0.0053	7^d^	8^d^	20^c^	1
	miR-449a	2,753	0.0071	6^d^	7^d^	20^c^	2
	miR-22-5p	2,598	0.0008	6^d^	8^d^	13^c^	1
	miR-342	3,164,347	0.0053	9,972^c^	10,024^c^	5,780^d^	600
	miR-378b	3,734	0.0017	18^c^	21^c^	8^d^	1

^a^ SER = Serum; LIV = Liver, SPL = Spleen; MLN = Mesenteric lymph node; TBLN = Tracheal-bronchial lymph node; and THY = Thymus.

^b^ CT = Control group; MB = Group challenged with M. bovis; CO = Co-infected group.

^c, d, e,^ Means (RPM) without a common superscript within row are statistically different (P< 0.01)

There were significant differences in miRNA counts among treatments for non-lymphoid tissues, as is the case for SER and LIV (Table 2). The CO group had the greatest number of copies for miR-1343-3p in SER, when compared to the CT and MB groups. In LIV, the CT and MB groups had the greatest number of copies for miR-382, and miR-154b, when compared to the CO group. The CT group had the greatest number of copies for miR-493 in LIV, when compared to the MB and CO groups.

Expression among treatments was similar for the seven significant (P< 0.01) miRNAs in SPL (Table 2). For all miRNAs, their expression in the CO group was lower, compared to the CT and MB groups. The CT and MB groups had similar expression of each miRNA and had the greatest number of copies.

In MLN, only miR-147 and miR-378 were significant (Table 2). For both miRNAs, the CT group had the greatest number of copies, when compared to the MB and the CO groups.

There were 21 miRNAs with differences in expression in TBLN (Table 2). In fourteen of these miRNAs, the CT group had the greatest number of copies, when compared to the MB and the CO groups. In the other seven miRNAs, the CO group had the greatest number of copies when compared to the CT and MB groups, except for miR-6529a and miR-96. For these miRNAs, the MB group had the greatest counts, compared to the CT and CO groups.

Table 2 shows the six miRNAs that had significant differences among treatments in THY. In four of the six miRNAs, the CO group had higher count numbers of each miRNA, compared to the CT and the MB groups. Conversely, for miR-342 and miR-378b, the CT and MB groups had the greatest number of counts, compared to the CO. For all miRNAs, their expression in the CO group was different from the CT and MB groups.

Two significant miRNAs were significant (P< 0.01) in two tissues (Table 2). MiR-378b was significant in SPL and in THY. In SPL and THY, the CO group had the least count number of miRNA sequences when compared to the CT and the MB groups. MiR-147 was significant in MLN and in THY. In MLN, this miRNA had the greatest count numbers in the CT group compared to MB and CO; however, in THY, the greatest count numbers were observed in the CO group. There were differences in expression of miR-147 depending on the expressed tissues.

### Nominally significant miRNAs in additional tissues

Fifteen significant miRNAs (P< 0.01) in one tissue were nominally significant (P< 0.05) in one or more of the other tissues (Table 3). Of these miRNAs, three were significant in one tissue, and nominally significantly in two additional tissues. MiR-409a was significant in TBLN, and nominally significant in LIV and SPL. MiR-382 was significant in LIV, and nominally significant in SPL and TBLN. MiR-378b was significant in SPL and THY, and nominally significant in MLN and TBLN. There were ten additional miRNAs that were significant in one, and nominally significant in another tissue. MiR-147 was the only miRNA that was significant in two tissues (MLN and THY), and nominally significant in TBLN.

**Table 3:** Normalized means (reads per million) per treatment of microRNAs that were identified as significant (P< 0.01) in one tissue, and were nominally significant (P< 0.05) in a different tissue.

					Treatment^b^			
Tissue^a^	miRNA	N	P-value	CT	MB	CO	SEM	Significant in tissue^a^
LIV	miR-409a	12166	0.042	173^c^	151^c^	93^d^	16	TBLN
	miR-421	1362	0.021	19^c^	15^d^	12^d^	1	SPL
SPL	miR-378	1016784	0.030	5317^c^	4860^c^	3937^d^	260	MLN
	miR-224	11318	0.018	58^c^	59^c^	36^d^	4	TBLN
	miR-409a	48686	0.033	277^c^	274^c^	136^d^	31	TBLN
	miR-382	16741	0.028	84^c^^,^^d^	102^c^	49^d^	10	LIV
	miR-411a	385779	0.017	1872^c^^,^^d^	2243^c^	1277^d^	160	TBLN
MLN	miR-381	68543	0.040	434^c^	198^d^	231^d^	47	TBLN
	miR-378b	2516	0.023	12^c^	10^c^^,^^d^	7^d^	1	SPL, THY
	miR-342	739542	0.034	2536^d^	3174^c^	2554^d^	145	THY
TBLN	miR-382	4110	0.015	32^c^	13^d^	11^d^	3	LIV
	miR-30f	60817	0.049	443^c^	197^d^	196^d^	55	SPL
	miR-378b	3972	0.038	35^c^	13^d^	8^d^	5	SPL, THY
	miR-147	5148	0.034	17^d^	17^d^	36^c^	4	MLN, THY
THY	miR-154b	2907	0.015	16^c^	16^c^	8^d^	1	LIV
	miR-30d	5511572	0.031	28845^c^	24436^c^	12625^d^		SPL
	miR-125b	1648235	0.032	4604^d^	4142^d^	11041^c^	1450	TBLN
	miR-29d-5p	11421	0.035	40^d^	33^d^	75^c^	9	TBLN

^a^ LIV = Liver, SPL = Spleen; MLN = Mesenteric lymph node; TBLN = Tracheal-bronchial lymph node; and THY = Thymus.

^b^ CT = Control group; MB = Group challenged with M. bovis; CO = Co-infected group.

^c, d^ Means (Reads Per Million) without a common superscript within row are statistically different (P< 0.01).

S1 Table shows the count for each miRNA in each tissue. There are tissue-specific differences in expression of miRNAs. That is, there are more than 1,000 counts for 1 tissue and fewer than 1,000 for the remaining tissues. As examples, bta-miR-141 is expressed mostly in THY, given the count for this tissue is 37,128, while the count is fewer than 1,000 for all other tissues. A similar pattern was identified for bta-miR-196a which is expressed in MLN (n = 9,147), bta-miR-205 which is expressed in THY (n = 317,977), bta-miR-2457 (n = 16,749), bta-miR-296-5p (n = 15,722), and bta-miR-323 (n = 19,394), which are expressed in WBC. There are additional miRNAs that appear to be tissue-specific; however, the difference among tissues is less dramatic.

## Discussion

It has been established that animals challenged with BVDV lead to a reduction in circulating lymphocytes (leukopenia), which is associated with immune suppression and increased susceptibility to secondary infections [9, 32]. Depletion of miRNAs was observed in LIV, SPL, MLN, and TBLN, in the CO group, compared to the CT and MB groups. It is possible the reduction in counts of miRNAs in these organs may be associated with the depletion of lymphocytes in cattle.

Animals challenged with BVDV have also shown a depletion of thymus in young calves [10]. However, the counts of miRNAs were greater in the CO group, when compared to the CT and MB groups. It is possible that BVDV requires the production of miRNAs in thymus to colonize the organism and inhibit its growth. Further studies would be needed to establish the role of miRNAs in the depletion of thymus in cattle.

Along with BVDV, *Mycoplasma bovis* also reduced the counts of miRNAs in TBLN in the CO group. The tracheal-bronchial lymph node is a relevant organ associated with the defense mechanism against respiratory pathogens due to be the nearest lymph node to the respiratory system. It is possible the reduction of miRNA counts is necessary for *M*. *bovis* to establish itself in lungs.

Tissues with the greatest number of significant and nominally significant miRNAs in differences in counts among treatments were identified were in TBLN, SPL, and THY. These organs are directly involved in the defense mechanism against bovine respiratory disease pathogens. Although MLN is part of the lymphatic system, it does not seem to be involved in the defense mechanism against pathogens that produce bovine respiratory disease.

The fewest significant miRNAs were identified in SER, and there were no significant differences in miRNA expression in WBC. Differences in expression of miRNAs was observed in tissues involved in the defense mechanism associated with respiratory diseases.

Patterns of miRNA expression were observed for the different tissues. For LIV and SPL, the CT and MB groups had the greatest counts of miRNAs. For all MLN and 68% of TBLN miRNAs, the CT group had the greatest counts; however, five miRNAs had the greatest count numbers in TBLN. For THY, the CO group had the greatest counts of miRNAs in four of the significant miRNAs. No pattern could be discerned in SER given that only miR-1343-3p was significant. It is probable that miRNA expression patterns are determined in each organ independently.

The significance of the expression of miRNAs in tissues from the lymphatic system are supported by the correlations among tissues. Correlations among LIV, MLN, TBLN, SPL, and THY, are high (r > 0.6), while correlations of SER and WBC, with the other tissues are low (r< 0.5). This seems to indicate that SER and WBC are inadequate predictors of miRNA expression when animals are challenged with BVDV or *M*. *bovis*. Correlations of MLN with LIV, TBLN, SPL, and THY, were high. Similarity of expression of miRNAs among tissues of the lymphatic system were greater than expected. Expression of miRNAs between MLN and TBLN seem to be similar, which is not surprising given that both organs have the same function within the lymphatic system. The expression of miRNAs between MLN, TBLN, and SPL were higher than expected (r > 0.85) but not surprising, given that all three of these tissues are part of the lymphatic system, and should be involved when animals are challenged with BVDV or *M*. *bovis*.

Serum and WBC should not be used to identify miRNAs to be used as predictors for challenge with BVDV or *M*. *bovis*. It was determined that miRNAs in SER comprise between 0.4% and 1% of the small non-coding RNAs of serum [22, 23, 31]. In the present study they account for 0.3% of the total miRNAs, when compared to the other tissues. This, and the low correlations between SER and WBC with the other tissues seem to indicate they should not be used to monitor challenged animals with BVDV or *M*. *bovis*. Several reviews have indicated the potential of using circulating miRNAs to potentially diagnose infectious diseases in livestock [33–35]. Based on the results from this study, bovine respiratory disease due to BVDV or *M*. *bovis* should be excluded from the list of conditions in which circulating miRNAs may be useful as predictors of the challenge. It has been proposed that regulation of circulating miRNAs is dependent on the species and the pathogen involved in the disease [34]. Based on the results from this study, it is also dependent of the tissue to be evaluated. Little or no gain will be achieved by measuring circulating miRNAs to identify animals exposed to BVDV or *M*. *bovis*.

MiRNAs have been identified as being associated with cell types or function in previous studies. MiR-382 had the greatest counts in LIV, SPL and TBLN. This miRNA has been identified as being involved in the inhibition of cancer growth and migration in humans [36, 37], but no reference exists of its function in bovine. It is possible that miR-382 could be associated with defense mechanisms against respiratory diseases in bovine. In the MLN and THY, miR-147 had significantly increased expression and was nominally significant in TBLN. The greatest counts for this miRNA were observed in the CO group in TBLN and in THY, whereas in MLN it had the greatest counts in CT. MiR-147 has been previously identified as being highly expressed in bovine alveolar macrophages [38, 39]. Given this miRNA has been found to be upregulated by the Toll-like receptor signaling pathway [40], it is likely that it has a role in the immunological development against BVDV or *M*. *bovis* by the animal.

MiRNAs identified in the present study as having differences among treatments have also been recognized in other studies. MiR-125a was downregulated in cows with mastitis when compared to cows without mastitis [41], which was also observed in the present study in TBLN. MiR-200b was downregulated in cows with mastitis [41], and in the present study the CO group had the highest counts for this miRNA. When expression analysis was done in ovine lungs affected with Visna/Maedi virus, to identify miRNA expression profiles, miR-125b was downregulated [42]. In the present study, miR-125b was also downregulated in TBLN.

It has been proposed that miRNAs have the potential to be used as indicators of disease and could be used to develop intervention strategies to minimize the effect of the disease. However, based on the results from this study, the evaluation of miRNAs by themselves will produce limited gain in improving animal health. An alternative approach is now being developed in which miRNAs are being evaluated jointly with messenger RNA to establish the biological relationship, making use of network or integrated analysis [43–45]. By identifying which messenger is associated with each miRNA, it could be possible to develop a strategy to regulate the miRNA, which in turn could regulate the messenger RNA. It is likely this will produce meaningful results that can be used to fully exploit the expression of miRNAs.

MiRNAs were evaluated in seven different tissues of calves challenged in a coinfection study with BVDV and *M*. *bovis*. Significant miRNAs were observed in tissues from the lymphatic system. BVDV was associated with reduction of miRNAs in these tissues, and *M*. *bovis* reduced counts of miRNAs in only tracheal-bronchial lymph node. Most tissue had a unique pattern of expression for significant miRNAs. The correlation of miRNA expression among the lymphatic system tissues evaluated were high, whereas the correlation of miRNA expression from these tissues with circulating miRNAs was low or non-existent. The use of circulating miRNAs to assess disease condition or to develop intervention strategies to minimize respiratory diseases in cattle caused by BVDV or *M*. *bovis* will be of limited use unless a different approach is developed. It must be remembered this is only a snapshot of a single time following infection. A more detailed study with additional time points, following infection are necessary to obtain a better overview of the expression of miRNAs. By adding time points, it will be possible to determine the peak of expression of each miRNA.

## Supporting information

S1 Table Total number of copies by tissue for each miRNA. (XLSX)
